# Explainable machine learning for Alzheimer's disease characterization using small-sample EEG data

**DOI:** 10.3389/fnagi.2026.1900857

**Published:** 2026-09-07

**Authors:** Lang Shen, Wei Tong, Ye Zhao, Bicheng Wu, Peng Zhang, Jing Kan

**Affiliations:** 1 Laboratory of Intelligent Brain Neuroimaging, Peking University Advanced Institute of Information Technology (AIIT), Hangzhou, Zhejiang, China; 2 School of Statistics and Data Science, Zhejiang Gongshang University, Hangzhou, Zhejiang, China; 3 Department of Psychiatry, Affiliated Xiaoshan Hospital, Hangzhou Normal University, Hangzhou, China

**Keywords:** Alzheimer's disease, EEG microstates, Interpretable machine learning, nonlinear complexity, quantitative EEG, resting-state EEG, SHAP

## Abstract

Alzheimer's disease (AD) is associated with progressive cognitive decline and altered brain functional activity, yet objective and interpretable electrophysiological indicators remain insufficiently established. Resting-state electroencephalography (EEG) offers a low-cost and clinically accessible candidate, provided that the analysis is validated at the subject level and remains interpretable. This study evaluated an interpretable resting-state EEG framework for distinguishing AD patients from healthy control (HC) subjects. A total of 63 participants (36 AD and 27 HC) from a publicly available dataset were included. Subject-level spectral and nonlinear complexity features were extracted from 19 preprocessed scalp channels; missing-value imputation, feature screening, redundancy pruning, scaling, and model fitting were carried out within each fold of leave-one-subject-out cross-validation. Four linear classifiers were compared, and Ridge Logistic regression was retained for out-of-fold SHAP interpretation because of its balanced hard-label performance and direct compatibility with Linear SHAP. Ridge Logistic regression achieved an exploratory AUC of 0.912 (accuracy = 0.857, sensitivity = 0.778, specificity = 0.963) under a non-nested validation design. Across 30 independently balanced epoch resamples, mean AUC was 0.887 ± 0.020; using all accepted epochs yielded AUC = 0.917. SHAP analysis indicated that the classifier drew jointly on posterior α activity, frontal and temporal θ power, the θ/α ratio, and slow/fast ratio features. A classifier-independent microstate analysis revealed reduced putative Class B occurrence, increased putative Class C and Class D duration, and six FDR-corrected off-diagonal transition differences in AD; the three temporal effects persisted across repeated *K* = 4 initializations and matched *K* = 3–6 solutions. These findings suggest that resting-state EEG can provide non-invasive and interpretable information about AD-related functional alterations, pending validation in larger, independent cohorts.

## 1 Introduction

Alzheimer's disease (AD) is the most common cause of dementia in older adults, and its clinical and societal burden continues to grow with population aging. Early and accessible identification of AD is therefore of considerable practical importance, but commonly used diagnostic-support tools have important limitations. Brief cognitive instruments such as the Mini-Mental State Examination (MMSE) and the Montreal Cognitive Assessment (MoCA) are widely used in primary care, yet their performance can be influenced by education, language, and cultural background (Patel and Grossberg, 2024; Ramos-Henderson et al., 2025). Amyloid and tau PET imaging can visualize AD-related pathological changes, but its high cost, limited availability, and requirement for radioactive tracer injection restrict routine screening applications (Jamerlan et al., 2024; Meeker et al., 2024). Cerebrospinal fluid (CSF) biomarkers, including Aβ_42_, total tau, and phosphorylated tau, also provide valuable diagnostic information, yet their reliance on lumbar puncture limits acceptability and widespread use (Agnello et al., 2025). Against this background, resting-state electroencephalography (EEG) offers a complementary and clinically accessible perspective: it is non-invasive, relatively low-cost, and suitable for repeated recordings, and its high temporal resolution enables the characterization of fast neural dynamics (Alidadi et al., 2026; Akbar et al., 2025; Modir et al., 2023). Resting-state EEG is also sensitive to AD-related neurophysiological alterations, including spectral slowing with a shift from faster to slower rhythms (Modir et al., 2023; Simfukwe et al., 2025), reductions in signal complexity (Simfukwe et al., 2025; Cacciotti et al., 2024), and altered scalp topographic dynamics (Piton et al., 2026; Casas Osorio et al., 2025). These properties together have motivated growing interest in resting-state EEG as a promising source of information for AD-related assessment.

Previous studies have applied machine-learning methods to resting-state EEG for classifying AD and related cognitive disorders, using handcrafted EEG features or deep learning models (Ding et al., 2022; Watanabe et al., 2024; Jia et al., 2024). These studies suggest that EEG contains discriminative information for AD identification. For example, Ding et al. (2022) reported an AUC of approximately 0.80 using spectral, complexity, and connectivity features; Yang et al. (2024) obtained comparable performance using microstate features, while deep learning models have also been evaluated in multi-center EEG datasets (Watanabe et al., 2024). However, reported performance is difficult to compare across studies because it is highly sensitive to validation design, especially in small clinical cohorts. In small cohorts, cross-validation estimates can have wide error bars and may vary with partitioning choices (Varoquaux, 2018). This issue is particularly important in EEG studies because each subject typically contributes multiple epochs. If epochs from the same subject appear in both training and test folds, models may exploit subject-specific information and yield optimistic estimates rather than reflecting subject-level generalization (Brookshire et al., 2024; Del Pup et al., 2025). These concerns motivate fold-wise subject-level validation, in which feature selection, redundancy control, and model fitting are performed within each training fold.

Beyond validation design, interpretability remains a key issue in EEG-based AD classification. *Post-hoc* attribution methods such as Shapley additive explanations (SHAP) and local interpretable model-agnostic explanations (LIME) have been used to identify EEG features contributing to AD classification decisions and to relate model outputs to quantities such as band power or signal entropy (Viswan et al., 2024; Vimbi et al., 2024; Gutiérrez-de Pablo et al., 2025). However, these methods explain how a trained model uses its inputs; they do not by themselves establish whether the highlighted features reflect broader AD-related neurophysiological alterations. Their interpretive value is therefore strengthened when combined with leakage-controlled validation and supported by a classifier-independent analysis. EEG microstates provide one such complementary perspective: previous studies and meta-analytic evidence have reported altered microstate temporal organization in AD, and microstate-derived features have also been explored as inputs for AD classification (Piton et al., 2026; Yang et al., 2024). In the present study, microstate analysis was used not as an additional feature source for the classifier, but as a classifier-independent complementary dynamic analysis alongside the spectral and nonlinear complexity features used for model training. This design choice was motivated by three considerations. First, maintaining methodological independence between the classification and microstate analyses allows each to serve as a form of convergent evidence rather than being dependent on one another. Second, several microstate metrics (e.g., GEV and coverage) may share variance with spectral power features, and introducing additional correlated features into the selection pipeline could reduce the stability of the selected feature set in a small-sample setting (*N* = 63). Third, microstate analysis characterizes global topographic dynamics, whereas spectral and complexity features capture local channel-level activity, and these two perspectives address complementary aspects of EEG signals.

The present study addresses these gaps by evaluating an interpretable resting-state EEG framework for distinguishing AD patients from HC subjects in the publicly available AHEPA dataset (OpenNeuro ds004504; Miltiadous et al., 2023), comprising 63 participants. Three design choices follow directly from the considerations above. First, the framework uses subject-level spectral and nonlinear complexity features extracted from 19 scalp channels, and classification is based on a small set of linear models to maintain stability and interpretability. Second, feature selection, redundancy control, and model fitting are performed within each fold of leave-one-subject-out cross-validation, so that reported performance reflects subject-level generalization while minimizing information leakage. Third, model explanation is performed in an out-of-fold manner using SHAP, aligning the explanation procedure with the validation strategy used for performance evaluation; in parallel, EEG microstate analysis is conducted as a classifier-independent dynamic analysis of resting-state topographic organization. Together, these design choices provide an interpretable and leakage-controlled framework for exploratory EEG-based AD characterization in a public, single-site cohort.

## 2 Materials and methods

**Figure 1:**
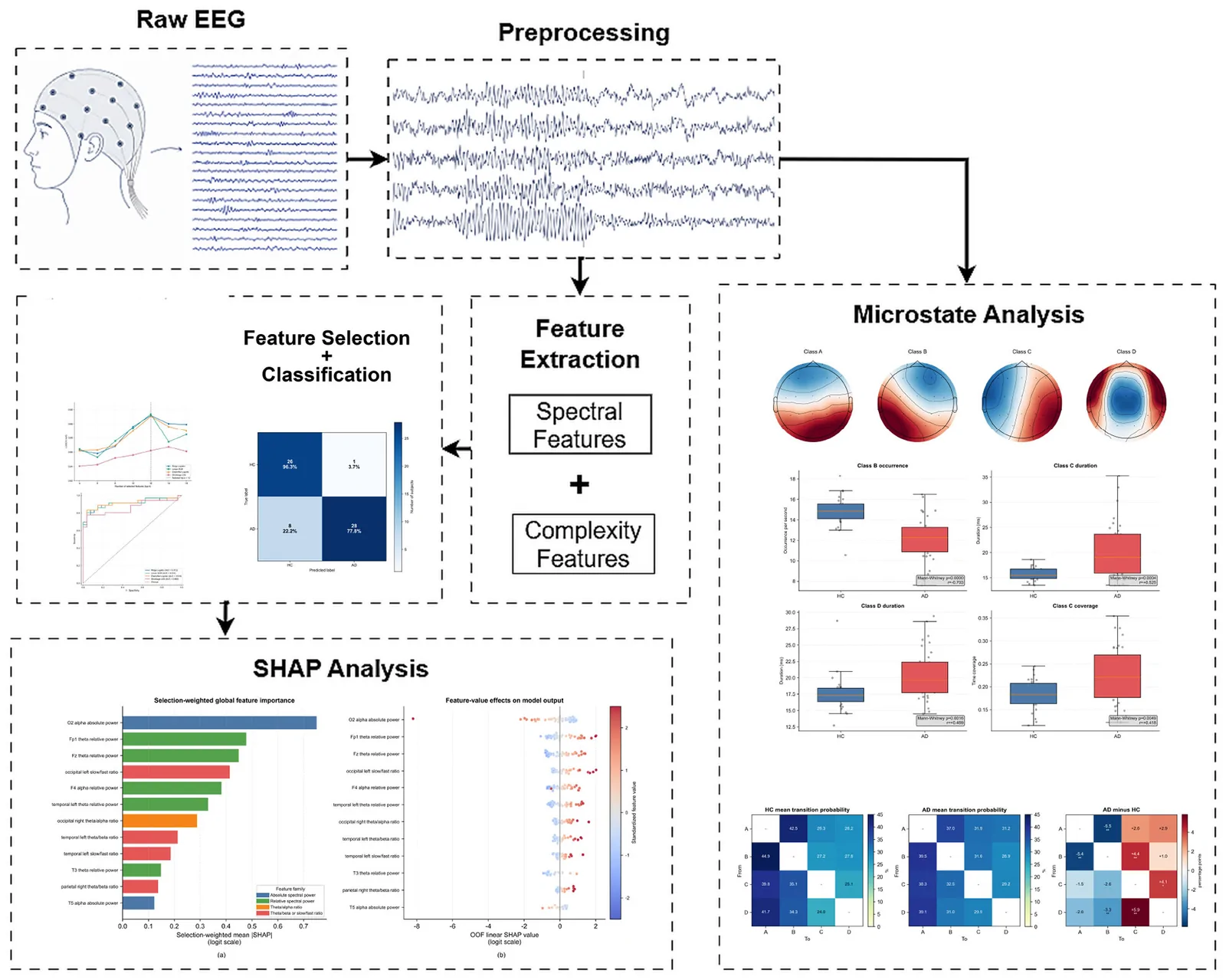
Overview of the proposed interpretable EEG framework for distinguishing Alzheimer's disease (AD) from healthy control (HC) subjects. Resting-state EEG recordings were first processed through a unified preprocessing pipeline. The primary classification pipeline extracted subject-level spectral and nonlinear complexity features, followed by fold-wise feature selection, linear classification under leave-one-subject-out cross-validation, and out-of-fold SHAP-based model explanation. In parallel, microstate analysis was performed as a complementary dynamic analysis to characterize temporal parameters and transition probabilities.

The overall analysis pipeline of the proposed interpretable EEG framework for Alzheimer's disease identification is summarized in Figure 1. Resting-state EEG recordings were first processed through a unified preprocessing pipeline, after which two complementary analyses were performed in parallel. The primary classification pipeline extracted subject-level spectral and nonlinear complexity features, followed by fold-wise feature selection, linear classification under leave-one-subject-out cross-validation, and out-of-fold SHAP-based model explanation. In parallel, microstate analysis was performed to characterize the temporal organization of resting-state scalp topographies as a complementary dynamic analysis, independent of the classification model. The following subsections describe each step in detail.

### 2.1 Participants and EEG data

This study used the publicly available AHEPA dataset (OpenNeuro accession ds004504; Miltiadous et al., 2023), which contains resting-state eyes-closed EEG recordings from 88 subjects acquired at the 2nd Department of Neurology of AHEPA General University Hospital of Thessaloniki, Greece. The study was approved by the Scientific and Ethics Committee of AHEPA University Hospital (protocol no. 142/12-04-2023). The dataset is distributed under the CC0 license.

For the present analysis, subjects with Alzheimer's disease (AD, Group “A”) and healthy controls (HC, Group “C”) were retained; subjects with frontotemporal dementia (Group “F”) were excluded. Two HC subjects were excluded because the corresponding derivative files could not be read, yielding a final analytic cohort of 63 subjects: 36 AD patients and 27 HC subjects. The demographic characteristics are summarized in Table 1. The AD and HC groups did not differ significantly in age (Mann–Whitney U test, *p* = 0.383) but differed in sex distribution (Fisher's exact test, *p* = 0.026). The Mini-Mental State Examination (MMSE) was available for all subjects; mean MMSE was 17.75 ± 4.50 in the AD group and 30.00 ± 0.00 in the HC group.

**Table 1:** Demographic characteristics of the final analytic cohort (AHEPA dataset).

Variable	AD (*n* = 36)	HC (*n* = 27)	*p*-value
Sex (male/female)	12/24	18/11	0.026
Age (years, mean ± SD)	66.4 ± 7.9	67.9 ± 5.4	0.383
MMSE (mean ± SD)	17.75 ± 4.50	30.00 ± 0.00	<0.001

AD, Alzheimer's disease; HC, healthy control; SD, standard deviation; MMSE, Mini-Mental State Examination score (range 0–30). The *p*-value for sex was calculated using Fisher's exact test, and *p*-values for age and MMSE were calculated using the Mann–Whitney U test.

Patients in the AD group met the NIA-AA clinical criteria for probable Alzheimer's disease (McKhann et al., 2011), with a mean disease duration of approximately 25 months (IQR 24–28.5 months). The diagnosis was based on clinical criteria without biomarker confirmation via PET, CSF, or MRI; therefore, the present findings pertain to clinically diagnosed probable AD rather than pathologically confirmed AD. The mean MMSE of 17.75 indicates moderate cognitive impairment in the AD group. HC subjects had an MMSE of 30, indicating no cognitive impairment, and no documented history of neurological or psychiatric disorders.

The EEG recordings were acquired using a Nihon Kohden EEG 2100 clinical device with 19 scalp electrodes (Fp1, Fp2, F7, F3, Fz, F4, F8, T3, C3, Cz, C4, T4, T5, P3, Pz, P4, T6, O1, and O2) placed according to the 10–20 international system, plus two reference electrodes (A1 and A2) on the mastoids. Recordings were performed with participants in a sitting position with eyes closed. The sampling rate was 500 Hz with 10 μV/mm resolution, and skin impedance was ensured to be below 5 kΩ before each recording. The referential montage using Cz as the common reference was used. Each recording lasted approximately 13.5 min for the AD group (range 5.1–21.3) and 13.8 minutes for the HC group (range 12.5–16.5). Following acquisition, the EEG recordings were exported to EEGLAB .set format. The public record does not provide standardized participant instructions beyond eyes-closed recording, wakefulness monitoring, individual recording times, or the identities and blinding status of manual raters; these protocol details could therefore not be further assessed here.

**Figure 2:**
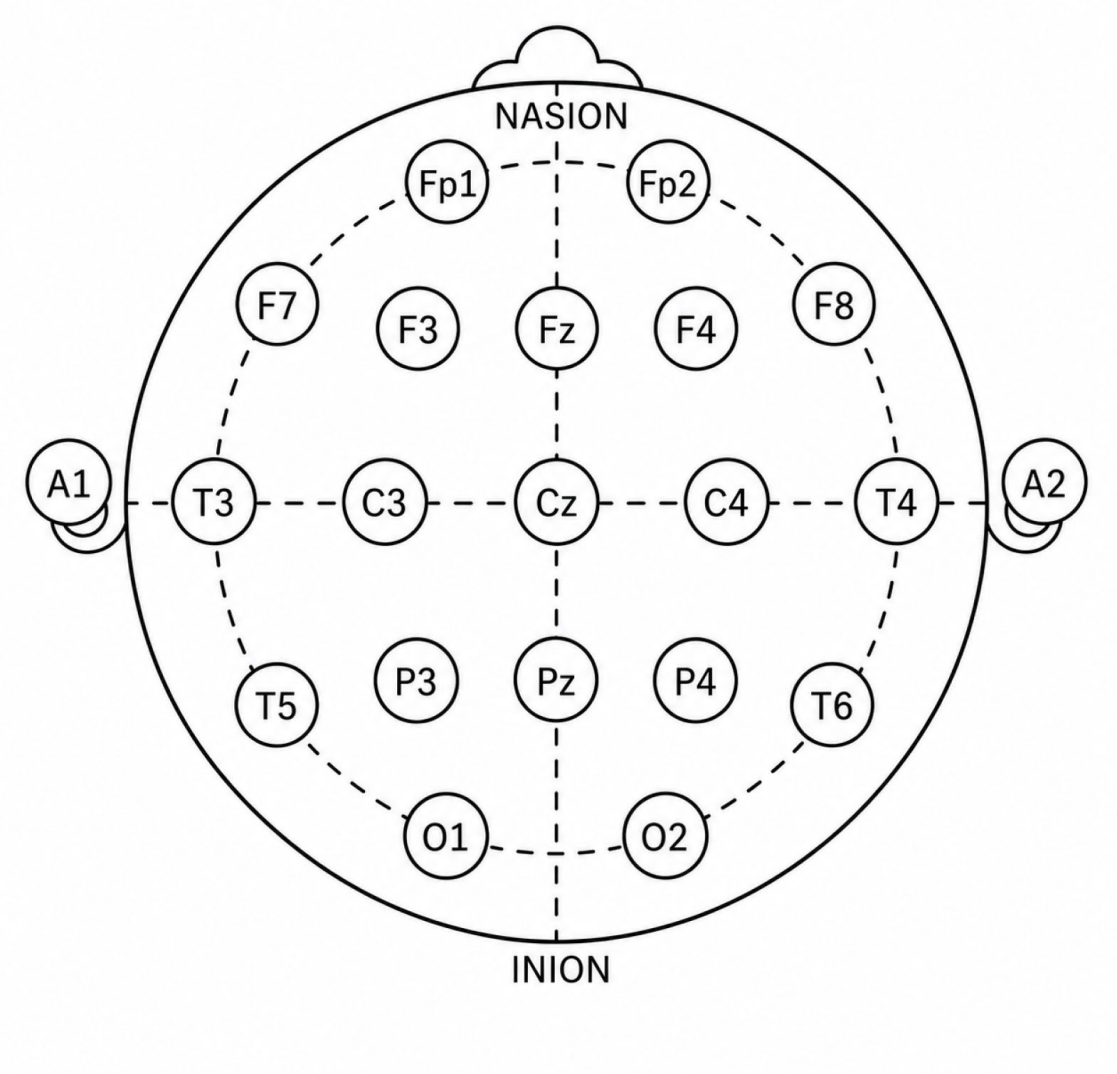
Layout of the retained 19 scalp EEG channels used in the analysis. The montage follows the conventional 10–20 nomenclature and includes Fp1, Fp2, F3, F4, C3, C4, P3, P4, O1, O2, F7, F8, T3, T4, T5, T6, Fz, Cz, and Pz. Channel Oz was not available in the AHEPA montage.

For the present study, we used the preprocessed derivatives provided with the AHEPA dataset. The preprocessing pipeline is described in detail by Miltiadous et al. (2023). Briefly, a Butterworth band-pass filter 0.5–45 Hz was applied, signals were re-referenced to the A1–A2 linked-mastoid reference, Artifact Subspace Reconstruction (ASR) was applied with a conservative 0.5-second window standard deviation cutoff of 17, and Independent Component Analysis (ICA; RunICA algorithm) was performed with components classified as eye or jaw artifacts by the ICLabel routine automatically rejected. To ensure consistency across subjects, a common set of 19 scalp EEG channels was retained for analysis: Fp1, Fp2, F3, F4, C3, C4, P3, P4, O1, O2, F7, F8, T3, T4, T5, T6, Fz, Cz, and Pz. Channel Oz was not present in the AHEPA montage. The spatial layout is shown in Figure 2.

### 2.2 EEG preprocessing

The AHEPA dataset provides preprocessed derivatives in which ASR and ICA-based artifact removal have already been applied (Miltiadous et al., 2023). For the present analysis, 19 scalp EEG channels (Fp1, Fp2, F3, F4, C3, C4, P3, P4, O1, O2, F7, F8, T3, T4, T5, T6, Fz, Cz, and Pz) were retained from the preprocessed .set files. All recordings were originally sampled at 500 Hz. Each recording was re-referenced to the common average reference, band-pass filtered between 1 and 40 Hz (FIR filter, Hamming window), and resampled to 100 Hz. To exclude unstable initial recording states, the first 60 s of each recording were discarded. The remaining continuous signals were segmented into non-overlapping 5 s epochs.

Because the AHEPA derivatives had already undergone ASR and ICA-based artifact rejection, epoch-level quality control resulted in minimal further rejection. Peak-to-peak amplitude thresholds of 500 μV (extreme), 300 μV (multi-channel), and 150 μV (across-channel median) were applied for consistency; fewer than 1% of epochs were rejected across the analytic cohort (Supplementary Table S1). To balance subject contributions while avoiding amplitude-biased epoch selection, the primary analysis retained a random sample without replacement (fixed seed 2026) of at most 60 accepted epochs per subject. One subject (sub-003) retained 49 epochs; the other 62 subjects retained 60 epochs. The mean usable recording duration after discarding the first 60 s was approximately 9.5 min per subject. Epoch-sampling sensitivity analyses recomputed the complete feature-extraction and LOOCV pipeline using all accepted epochs and 30 independently balanced random samples (seeds 2026–2055).

### 2.3 EEG feature extraction

Based on the preprocessed clean epochs, EEG features were extracted from two complementary perspectives: spectral rhythmic activity and nonlinear signal complexity. All features were ultimately aggregated to the subject level, yielding a single feature vector per subject for subsequent analysis.

#### 2.3.1 Spectral features

The power spectral density (PSD) was estimated for each clean epoch using Welch's method (Welch, 1967; Zheng et al., 2023). Given the 100 Hz sampling rate after preprocessing, a 256-point segment corresponded to a 2.56 s analysis window, with 50% overlap between adjacent segments. Let ${\hat{S}}_{c}\left(f\right)$ denote the epoch-averaged PSD of channel *c* for a given subject, and let ℱ_*b*_ denote the frequency bins within band *b*. Four conventional frequency bands were defined: delta (1–4 Hz), theta (4–8 Hz), alpha (8–13 Hz), and beta (13–30 Hz). The absolute power of channel *c* in band *b* was calculated as


$$\begin{array}{c} P_{c,b}=\int_{F_{b}}{\hat{S}}_{c}\left(f\right)df, \end{array}$$ (1)

which was numerically approximated via trapezoidal integration over the corresponding frequency bins. The relative power was then defined as


$$\begin{array}{c} {\tilde{P}}_{c,b}=\frac{P_{c,b}}{\int_{1}^{30}{\hat{S}}_{c}\left(f\right)df}, \end{array}$$ (2)

where the 1–30 Hz range served as the total spectral power reference. Channel-level absolute and relative powers were further aggregated into regional spectral features by averaging within predefined scalp regions: left frontal (Fp1, F3, F7) and right frontal (Fp2, F4, F8); left and right temporal (T3, T5, F7 and T4, T6, F8); central (C3, C4); parietal (P3, P4); and occipital (O1, O2). Midline electrodes (Fz, Cz, Pz) were retained as midline summary features, and a global summary was additionally computed across all 19 EEG channels.

To characterize EEG slowing, three standard ratio features were derived from the regional relative powers: the theta/alpha ratio (TAR), the theta/beta ratio (TBR), and the slow/fast wave ratio (SFR), defined respectively as


$$\begin{array}{c} \text{TA}{\text{R}}_{r}=\frac{{\tilde{P}}_{r,\theta }}{{\tilde{P}}_{r,\alpha }},\text{ TB}{\text{R}}_{r}=\frac{{\tilde{P}}_{r,\theta }}{{\tilde{P}}_{r,\beta }},\text{ SF}{\text{R}}_{r}=\frac{{\tilde{P}}_{r,\delta }+{\tilde{P}}_{r,\theta }}{{\tilde{P}}_{r,\alpha }+{\tilde{P}}_{r,\beta }}, \end{array}$$ (3)

where *r* denotes the scalp region. For each of the five homologous left–right region pairs (frontal, central, temporal, parietal, and occipital), a hemispheric asymmetry index (AI) was further calculated from the regional relative powers:


$$\begin{array}{c} \text{A}{\text{I}}_{r,b}=\frac{{\tilde{P}}_{R\left(r\right),b}-{\tilde{P}}_{L\left(r\right),b}}{{\tilde{P}}_{R\left(r\right),b}+{\tilde{P}}_{L\left(r\right),b}}, \end{array}$$ (4)

where ${\tilde{P}}_{L\left(r\right),b}$ and ${\tilde{P}}_{R\left(r\right),b}$ denote the relative powers of the left and right homologous regions in band *b*, respectively.

#### 2.3.2 Nonlinear complexity features

To capture the temporal irregularity and amplitude-distribution properties of the EEG signals, two nonlinear complexity measures were computed: permutation entropy (PE) and differential entropy (DE). Prior to complexity analysis, all EEG signals were amplitude-scaled to microvolts (μV).

Permutation entropy was computed for each epoch and channel with an embedding dimension *m* = 3 and a time delay τ = 1 (Bandt and Pompe, 2002; Edmonds, 2026). Given the probability distribution *p*(π) of ordinal pattern π, the normalized PE was defined as


$$\begin{array}{c} \text{PE}=-\frac{1}{\log\left(m!\right)}\underset{\pi }{\sum }p\left(\pi \right)\log p\left(\pi \right). \end{array}$$ (5)

Differential entropy was computed under a Gaussian assumption for each epoch-level signal segment: for a segment with variance σ^2^,


$$\begin{array}{c} \text{DE}=\frac{1}{2}\log\left(2\pi e\sigma^{2}\right). \end{array}$$ (6)

For each channel, the epoch-level PE and DE values were averaged across epochs to obtain subject-level channel features. Regional complexity features were then derived by averaging these channel-level metrics within the same predefined scalp regions used for the spectral analysis. Left–right asymmetry indices for PE and DE were also computed across the five homologous region pairs using the same normalized difference form as for spectral asymmetry.

#### 2.3.3 Final candidate features

The complete subject-level feature matrix comprised 301 spectral features and 72 nonlinear complexity features, yielding a total of 373 candidate EEG features that served as the input pool for the subsequent feature selection and classification analyses.

### 2.4 Feature selection and classification framework

All classification analyses were conducted at the subject level. After feature extraction, each subject was represented by a single feature vector with the corresponding diagnostic label (AD or HC), and the candidate feature pool consisted of 373 features (301 spectral and 72 nonlinear complexity features). Epochs were used only for within-subject feature computation and aggregation, and were not treated as independent samples for classification.

To avoid information leakage, feature selection was performed independently within each training fold. Within the training set of each fold, missing values were imputed using the training-fold median, and features with zero variance were removed. The remaining features were then ranked according to their univariate discriminative ability, measured by the absolute deviation of the univariate AUC from chance level, |AUC − 0.5|. To reduce redundancy among highly correlated features, the ranked features were sequentially screened using Spearman correlation: a candidate feature was retained only if its absolute correlation with all previously selected features was below a predefined threshold of 0.90. The number of retained features *k* was treated as a tunable hyperparameter and evaluated through a prespecified sensitivity analysis over the grid {4, 6, 8, 10, 12, 14, 16}. The global LOOCV profile was used to choose the unified setting *k* = 12 and to retain Ridge Logistic for SHAP visualization because of its balanced hard-label performance and direct compatibility with Linear SHAP. These global choices were not made through nested inner cross-validation. The reported AUC therefore represents an exploratory estimate that may be optimistically biased relative to independent-sample performance; the use of a non-nested selection procedure should be taken into account when interpreting the classification results.

Based on the selected feature subset in each training fold, four linear classifiers were evaluated: ridge logistic regression, elastic-net logistic regression, linear support vector machine (SVM), and shrinkage linear discriminant analysis (LDA) (Friedman et al., 2010; Cortes and Vapnik, 1995; Kim et al., 2024; Yang et al., 2024). For classifiers supporting class weights, balanced class weights were applied within the training fold. Feature scaling and model fitting were performed using the training data only, and the fitted transformations were subsequently applied to the held-out subject. Overall classification performance was assessed using leave-one-subject-out cross-validation, in which each of the 63 subjects was held out once for evaluation while the remaining 62 subjects served as the training set.

#### 2.4.1 Epoch-sampling sensitivity analysis

To quantify the dependence of the subject-level results on the random epoch sample, all 373 features were recomputed for each of 30 independently balanced random samples (seeds 2026–2055) and for an analysis using every accepted epoch. For each feature matrix, the full Ridge Logistic LOOCV pipeline was rerun, including fold-wise imputation, feature ranking, redundancy pruning, scaling, and model fitting. We summarized the distribution of AUC, accuracy, sensitivity, specificity, precision, and F1 score across random samples. For feature selection, we additionally summarized the selection frequency of each feature across the 30 resampled analyses and the pairwise Jaccard overlap of the sets of features selected at least once.

### 2.5 Out-of-fold SHAP analysis

To obtain model explanations consistent with the leave-one-subject-out validation procedure, SHAP analysis was performed in an out-of-fold manner. Within each LOOCV fold, missing-value imputation, univariate feature ranking, Spearman-based redundancy pruning, feature standardization, and ridge logistic regression fitting were performed using the training subjects only. The standardized selected features from the corresponding training fold were used as the background data for the Linear SHAP explainer, and SHAP values were computed exclusively for the held-out subject. This procedure ensured that each subject's explanation was derived from a model trained without that subject, preserving the same out-of-fold principle used for performance evaluation.

SHAP provides an additive feature-attribution framework that decomposes a model output into a baseline value and the contributions of individual features:


$$\begin{array}{c} g\left\{f\left({\text{x}}_{i}\right)\right\}=\phi_{0}+\underset{j\in S_{i}}{\sum }\phi_{ij}, \end{array}$$ (7)

where *f*(**x**_*i*_) denotes the model prediction for subject *i*, *g*(·) denotes the output scale on which the SHAP values are additive, ϕ_0_ is the expected model output under the background distribution, ϕ_*ij*_ is the SHAP value of feature *j* for subject *i*, and 𝒮_*i*_ denotes the feature set used by the fold-specific model corresponding to subject *i*. In this study, SHAP values were interpreted as additive contributions to the fold-specific ridge logistic model output. Positive SHAP values pushed the model output toward AD, whereas negative SHAP values pushed it toward HC.

Since the selected feature set 𝒮_*i*_ could vary across LOOCV folds, SHAP values were stored and summarized in long format. Features not selected in a given fold were treated as absent from that fold-specific model rather than assigned a SHAP value of zero. Across all held-out subjects, feature-level explanations were summarized by four complementary statistics: the selection frequency across folds, the mean absolute SHAP value when selected, the mean signed SHAP value when selected, and the average direction of feature contribution. Selection frequency and mean SHAP values were reported separately to allow independent assessment of feature stability and effect size. A selection-weighted global importance score, defined as the product of the selection frequency and the mean absolute SHAP value when selected, was used to rank features by their combined frequency and influence across folds.

### 2.6 Microstate analysis

Microstate analysis was performed on the preprocessed 19-channel EEG epochs to characterize the temporal organization of resting-state scalp topographies (Michel and Koenig, 2018; Piton et al., 2026; Chang, 2025). This analysis was conducted independently of the classification pipeline, and microstate-derived features were not used for classifier training. For each subject, the global field power (GFP) was calculated as the spatial standard deviation across channels at each time point, and topographies at GFP peaks were extracted as they correspond to stable and high-signal scalp configurations. Each GFP peak map was then average-referenced and *L*_2_-normalized. To ensure equal subject contributions to the group-level template estimation, at most 1,000 GFP peak maps were retained per subject.

Group-level microstate templates were estimated by pooling the GFP peak maps across all subjects and applying polarity-invariant k-means clustering. A four-template solution (*K* = 4) was adopted for the main microstate analysis, based on prior literature (Michel and Koenig, 2018). The primary *K* = 4 initialization used seed 12,025 and explained 56.4% of the total GFP peak variance (unweighted GEV). The resulting templates were initially labeled as MS1–MS4 and sorted according to their cluster-level explained variance. For ease of reference, these templates are referred to as putative Classes A–D in order of descending explained variance. Because the A–D labels were assigned by variance ordering rather than by validated source-localization or spatial correlation with normative templates, they should be interpreted as provisional rather than as established canonical microstate classes.

To assess clustering robustness, we repeated polarity-invariant clustering with 30 independent initializations (seeds 2026–2055) at each of *K* = 3, 4, 5, 6, while retaining the same GFP-peak pool and convergence settings. For each *K*, candidate templates were aligned to a reference solution by Hungarian matching of absolute spatial correlations. We summarized template similarity and GEV across initializations. For *K* = 4, temporal group effects were recomputed for the primary solution and all 30 robustness solutions. For *K* = 3, 5, 6, each temporal effect was evaluated in the template having the highest absolute spatial correlation with its corresponding primary *K* = 4 topography.

The four group-level templates were then back-fitted to each subject's preprocessed EEG epochs. At each time point, the microstate label was assigned according to the highest absolute spatial correlation between the instantaneous scalp map and the group-level templates, in a polarity-invariant manner. Epoch boundaries were preserved so that segments were not bridged across discontinuous epochs. For each subject, the following microstate temporal metrics were computed: mean duration, occurrence rate, and coverage for each microstate class. Transition probabilities were additionally calculated between consecutive run-length segments within each 5-s epoch; transitions were not counted across epoch boundaries, and self-transitions were excluded by construction. For each origin class, the three off-diagonal transition counts were normalized by their row total. Group differences in temporal metrics were assessed using the Mann–Whitney U test (Mann and Whitney, 1947). The 12 off-diagonal transition probabilities were tested in the same way and adjusted using the Benjamini–Hochberg false-discovery-rate procedure. Effect sizes were reported using the rank-biserial correlation with the direction of group difference explicitly indicated.

### 2.7 Computational settings for reproducibility

All analyses were implemented in Python (version 3.11). Key software packages included scikit-learn (1.7.2), SciPy (1.16.3), MNE-Python (1.12.1), NumPy (2.3.5), Pandas (2.3.3), and Matplotlib (3.10). The following parameters were pre-specified unless otherwise indicated.

#### 2.7.1 Classification settings

Ridge logistic regression was fitted with penalty *L*_2_, regularization strength *C* = 1.0, solver liblinear, class weight balanced, maximum iterations 5,000, and random state 2026. ElasticNet logistic regression used penalty elasticnet, *C* = 1.0, *L*_1_ ratio = 0.5, solver saga, class weight balanced, maximum iterations 20,000, tolerance 10^−4^, and random state 2026. Linear SVM used *C* = 1.0, class weight balanced, maximum iterations 20,000, and random state 2026. Shrinkage LDA used solver lsqr with automatic shrinkage (Ledoit–Wolf).

#### 2.7.2 Feature selection settings

Univariate feature ranking used |AUC − 0.5| as the ranking metric. Spearman correlation redundancy pruning used a threshold of |ρ|≥0.90, with the top *N* = 120 ranked features preselected before pruning. The number of retained features *k* was evaluated over the prespecified grid {4, 6, 8, 10, 12, 14, 16}. The unified setting of *k* = 12 was selected from the global LOOCV performance profile (exploratory; not from nested inner cross-validation).

#### 2.7.3 Microstate settings

Polarity-invariant *k*-means clustering used up to 1,000 GFP peak maps per subject, a convergence tolerance of 10^−7^, and a maximum of 300 iterations. The primary temporal analysis used *K* = 4 and initialization seed 12,025. Robustness analyses used *K* = 3, 4, 5, 6 and 30 initializations per *K* (seeds 2026–2055), with templates aligned by Hungarian matching of absolute spatial correlations. The A–D labels were assigned in order of descending cluster-level explained variance; spatial correlation with canonical templates was not performed.

#### 2.7.4 Random seeds

Seed 2026 was used for the primary epoch sample, GFP-peak subsampling, and classifier random states. The primary *K* = 4 clustering initialization used seed 12,025. The epoch-sampling and microstate robustness analyses used the fixed seed sequence 2026–2055.

## 3 Results

The results are organized in five parts. An exploratory characterization of the candidate EEG feature space is presented first, providing visual context for the subsequent classification, model-explanation, and microstate analyses. Classification performance under leave-one-subject-out cross-validation and the corresponding out-of-fold SHAP explanations together form the core of the interpretable classification framework, followed by a confounder analysis examining demographic influences. Finally, the microstate analysis—conducted independently of the classifier—offers a separate window on resting-state topographic dynamics in AD.

### 3.1 Exploratory analysis of EEG-derived features

The candidate feature pool comprised 373 subject-level EEG features, including 301 spectral and 72 nonlinear complexity features. An exploratory characterization of this feature space was first carried out for descriptive visualization of group-level patterns; it was not used for model fitting, feature selection, or hyperparameter tuning.

**Figure 3:**
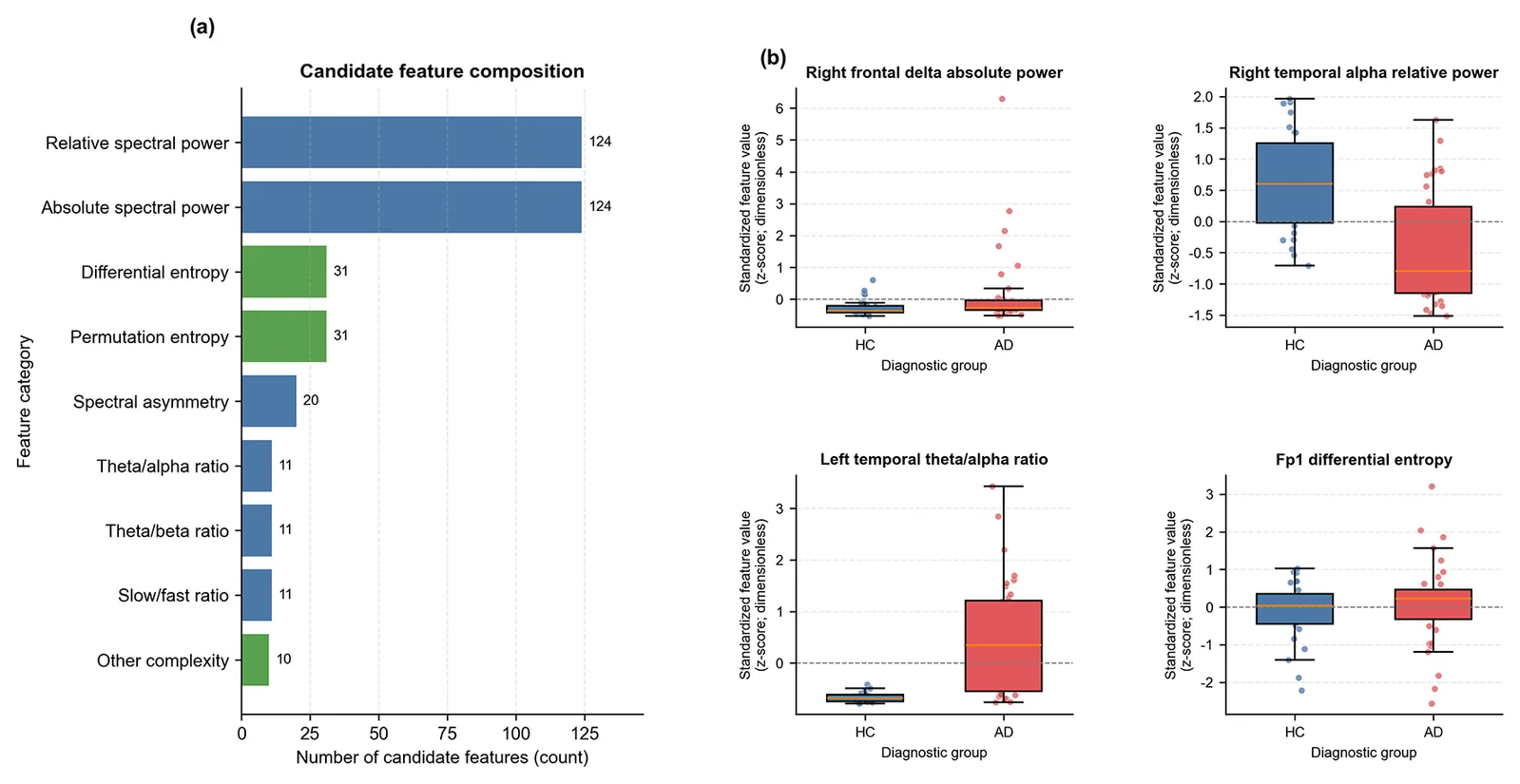
Exploratory characterization of the 373 subject-level EEG candidate features. **(a)** Composition of the candidate feature pool, color-coded by feature family: spectral features (blue), including absolute and relative power, theta/alpha, theta/beta, and slow/fast ratios, and hemispheric asymmetry; and nonlinear complexity features (green), including differential entropy and permutation entropy. The number of features in each subcategory is annotated next to the corresponding bar. **(b)** Group distributions of four representative individual features, illustrating visible AD–HC differences in spectral power (right frontal δ absolute power), relative power (right temporal α relative power), spectral ratio (left temporal θ/α ratio), and nonlinear complexity (Fp1 differential entropy). Boxplots show the median and interquartile range, overlaid with individual subject-level data points. Feature values were z-scored across all subjects for visualization. HC, healthy controls (blue); AD, Alzheimer's disease (orange). This figure was used only for exploratory visualization of group-level patterns and was not used for model fitting, feature selection, or hyperparameter tuning.

Figure 3a summarizes the composition of the candidate pool, which was dominated by spectral power features (absolute and relative power), with smaller contributions from spectral ratios, hemispheric asymmetry measures, and nonlinear complexity metrics (permutation entropy and differential entropy). Several individual features showed visible AD–HC differences at the group level (Figure 3b): right frontal δ absolute power and left temporal θ/α ratio were elevated in AD, right temporal α relative power was reduced, and Fp1 differential entropy was higher in AD than in HC. These descriptive observations preceded—and were independent of—the formal feature selection and model fitting, which were performed strictly within each LOOCV training fold to avoid information leakage.

### 3.2 Classification performance under leave-one-subject-out validation

Subject-level discriminative performance was assessed using leave-one-subject-out cross-validation (LOOCV). Within each fold, missing-value imputation, zero-variance filtering, univariate AUC-based ranking, Spearman-based redundancy pruning, feature standardization, and classifier fitting were carried out using the training subjects only, with the held-out subject reserved exclusively for evaluation.

**Figure 4:**
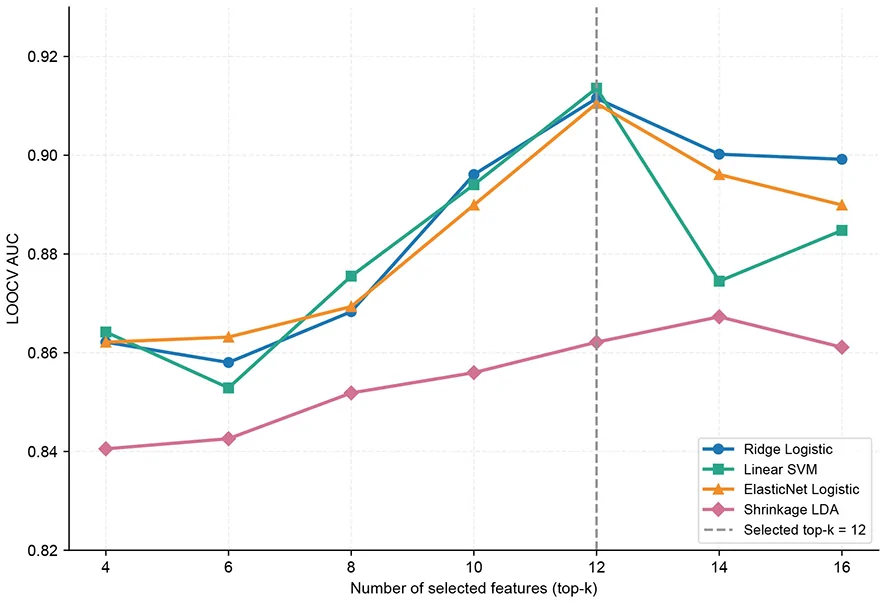
Top-*k* sensitivity analysis of the four linear classifiers under leave-one-subject-out cross-validation. The *x*-axis denotes the number of selected features (*k*) used by each fold-specific model, and the *y*-axis denotes the LOOCV AUC. Each curve represents one classifier: Ridge Logistic regression (blue circles), Linear support vector machine (green squares), ElasticNet Logistic regression (orange triangles), and Shrinkage linear discriminant analysis (pink diamonds). The vertical dashed line marks the unified setting of *k* = 12 adopted in all subsequent analyses.

The number of selected features *k* was treated as the first quantity to be calibrated. Figure 4 plots the LOOCV AUC of all four classifiers across the prespecified grid {4, 6, 8, 10, 12, 14, 16}. AUC rose substantially as *k* increased from 4 to the intermediate range of 8–12 for every classifier; beyond *k* = 12, performance plateaued and showed mild fluctuations, indicating that additional features began to bring in redundant or noisy information rather than further discriminative signal. Ridge Logistic reached its highest observed AUC at *k* = 12 (AUC = 0.912), with comparable values at *k* = 10 (AUC = 0.896) and *k* = 14 (AUC = 0.900), and the remaining classifiers were close to their respective maxima at the same value. Because this *k* value was identified from the global LOOCV profile rather than from a nested inner cross-validation loop, the reported AUC at *k* = 12 should be regarded as an exploratory upper-bound estimate rather than a validated performance level; without nested validation, the AUC is likely optimistic relative to independent-sample performance. Notably, adjacent values (*k* = 10 and *k* = 14) yielded comparable AUC values, suggesting that the overall performance level is not critically dependent on the exact choice of *k*. The unified setting of *k* = 12 was therefore adopted for all subsequent analyses.

At this unified setting (Table 2), LOOCV AUC ranged from 0.862 to 0.914 and classification accuracy from 0.810 to 0.857. Ridge Logistic achieved a competitive AUC (0.912) and also led in accuracy (0.857) and F1-score (0.862), with sensitivity 0.778, specificity 0.963, and precision 0.966. Linear SVM produced a slightly higher AUC (0.914) but with perfect specificity (1.000) at the expense of lower sensitivity (0.667), reflecting a more conservative decision boundary. ElasticNet Logistic performed similarly to Ridge Logistic (AUC = 0.910, accuracy = 0.857). Shrinkage LDA stood out with the lowest AUC (0.862) but identical hard-label accuracy to Ridge Logistic (0.857). Ridge Logistic was retained for the subsequent visualization and SHAP-based interpretation because of its competitive classification performance, balanced sensitivity-specificity profile, and its direct compatibility with the Linear SHAP framework.

**Table 2:** Classification performance of four linear classifiers under leave-one-subject-out validation at *k* = 12.

Model	AUC	ACC	SEN	SPE	PRE	F1
Ridge Logistic	0.912	**0.857**	0.778	0.963	**0.966**	**0.862**
ElasticNet Logistic	0.910	**0.857**	0.778	0.963	**0.966**	**0.862**
Linear SVM	**0.914**	0.810	0.667	**1.000**	**1.000**	0.800
Shrinkage LDA	0.862	**0.857**	0.778	0.963	**0.966**	**0.862**

The best value in each metric column is highlighted in bold. AUC, area under the receiver operating characteristic curve; ACC, accuracy; SEN, sensitivity; SPE, specificity; PRE, precision; SVM, support vector machine; LDA, linear discriminant analysis. AD was treated as the positive class for sensitivity, precision, and F1-score. All steps of missing-value imputation, feature ranking, redundancy pruning, feature standardization, and classifier fitting were performed independently within each leave-one-subject-out training fold to prevent information leakage.

**Figure 5:**
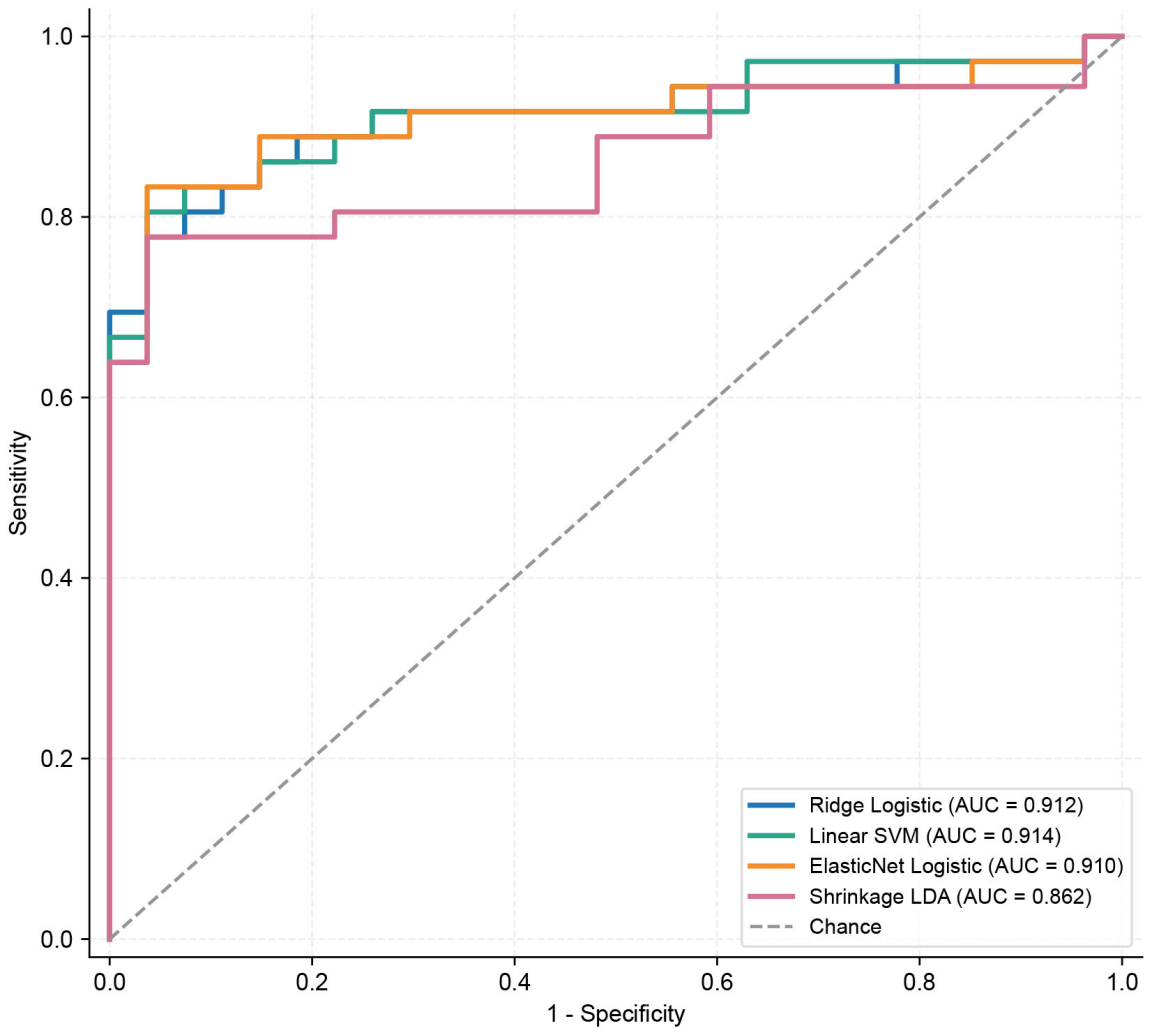
Receiver operating characteristic (ROC) curves of the four linear classifiers under leave-one-subject-out cross-validation at *k* = 12. The *x*-axis denotes 1 − specificity and the *y*-axis denotes sensitivity, with AD treated as the positive class. Each curve corresponds to one classifier, and the area under the curve (AUC) is reported in the legend. The diagonal dashed line indicates chance-level performance. The stepwise appearance of the curves reflects the discrete decision thresholds determined by the predicted scores of the 63 held-out subjects.

**Figure 6:**
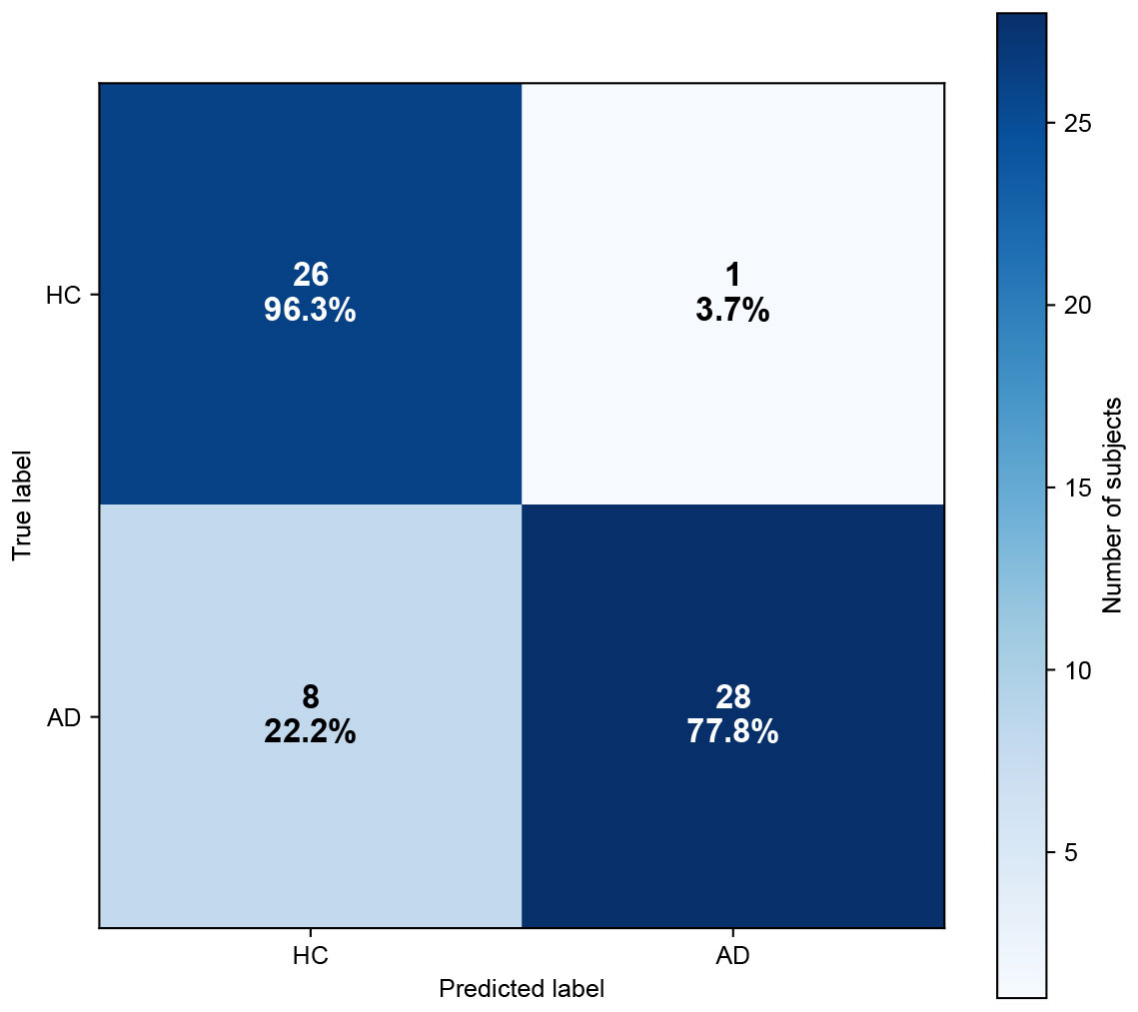
Confusion matrix of the Ridge Logistic model under leave-one-subject-out cross-validation at *k* = 12, with AD treated as the positive class. Rows correspond to true labels and columns correspond to predicted labels. Each cell shows the number of held-out subjects **(top)** and the corresponding row-normalized percentage **(bottom)**; the color intensity encodes the subject count, with darker shades indicating higher values. The model correctly classified 26 of 27 HC subjects (specificity = 96.3%) and 28 of 36 AD patients (sensitivity = 77.8%), corresponding to 1 false-positive and 8 false-negative subject-level predictions.

The corresponding ROC curves are displayed in Figure 5. To further examine the subject-level prediction behavior of the model selected for SHAP visualization, the confusion matrix of Ridge Logistic is shown in Figure 6. Under LOOCV, Ridge Logistic correctly classified 28 of 36 AD patients (sensitivity = 77.8%) and 26 of 27 HC subjects (specificity = 96.3%), yielding 8 false negatives and 1 false positive. The higher false-negative rate reflects the asymmetry of the AD/HC sample sizes (36 vs. 27) under balanced class weighting. Taken together, the comparable AUC across all four linear classifiers and the balanced subject-level prediction profile of Ridge Logistic indicate that the EEG-derived spectral and complexity features carried favorable discriminative information for AD–HC classification under strict subject-level validation, rather than reflecting performance specific to a single model architecture. To assess whether age or sex could have driven the classification performance, a demographic-only model (age + sex) was evaluated under the same LOOCV framework, yielding an AUC of 0.58, near chance level. Age and sex were not selected in any LOOCV fold of the combined (demographic + EEG) model, and the combined-model AUC was identical to the EEG-only AUC. Thus, including these variables did not materially improve discriminative performance in this LOOCV comparison.

### 3.3 Epoch-sampling sensitivity analysis

The primary epoch sample (seed 2026) reproduced the reported Ridge Logistic performance (AUC = 0.912; accuracy = 0.857). Across 30 independently balanced random epoch samples, the mean AUC was 0.887 ± 0.020 (range: 0.829–0.921), with mean accuracy 0.811 ± 0.024 (range: 0.762–0.857), sensitivity 0.747 ± 0.023, and specificity 0.896 ± 0.036. When all accepted epochs were used, AUC was 0.917 and accuracy was 0.841. Thus, classification performance remained above chance under both alternatives, but showed moderate dependence on the particular epochs sampled; the fixed-seed result should not be interpreted as a unique or definitive estimate. Across the random samples, O2 α absolute power, F7 θ absolute power, Fp1 θ relative power, and T3 θ relative power had the highest feature-selection frequencies (87%–95%). The mean pairwise Jaccard overlap among selected-feature sets was 0.535, indicating a subset of high-frequency features alongside variation in secondary selected features. Full resampling results are reported in Supplementary Table S3.

### 3.4 Out-of-fold SHAP-based model explanation

Beyond comparing models, we asked which EEG features the Ridge Logistic classifier actually relied on, and how those features pushed each individual prediction. This question was addressed with an out-of-fold SHAP analysis at the unified *k* = 12 setting. In each LOOCV fold, SHAP values were computed for the held-out subject from the corresponding fold-specific model, so that every subject's explanation came from a classifier trained without that subject. Because the 12 selected features could differ from fold to fold, feature importance was quantified in a selection-weighted manner, combining the mean absolute SHAP value (when a feature was selected) with its across-fold selection frequency.

**Figure 7:**
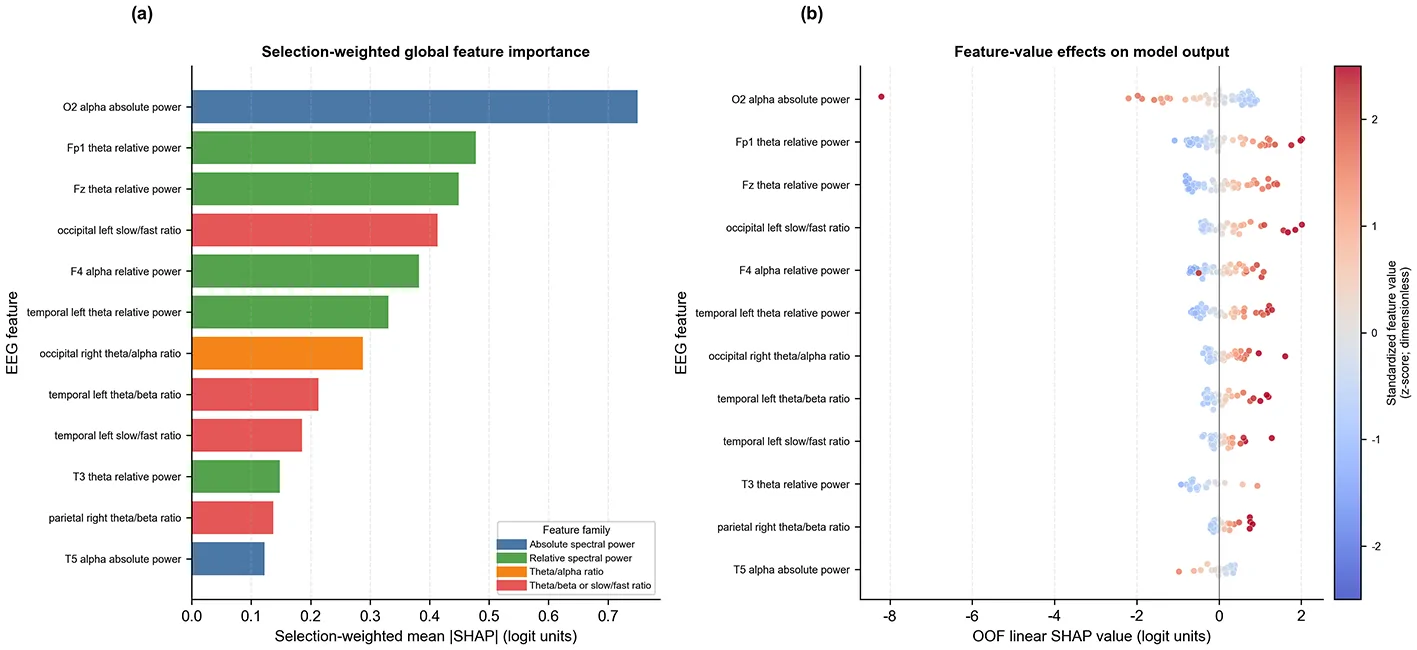
Out-of-fold SHAP-based explanation of the Ridge Logistic model under the unified setting of *k* = 12. **(a)** Selection-weighted global SHAP importance of the 12 features with the highest combined selection frequency and influence across leave-one-subject-out folds. The horizontal axis represents the mean absolute SHAP value (on the logit scale) weighted by the across-fold selection frequency. Bar colors denote the feature family: absolute spectral power (blue), relative spectral power (green), θ/α ratio (orange), and slow/fast ratio (red). **(b)** Out-of-fold Linear SHAP beeswarm plot for the same 12 features, sorted in the same order as in **(a)**. Each point represents the SHAP value of a feature for a single held-out subject under the corresponding fold-specific model. Positive SHAP values push the prediction toward AD, whereas negative values push it toward HC. Point color encodes the standardized feature value, with red indicating higher and blue indicating lower values.

Ranked by this selection-weighted importance, the 12 most influential features are shown in Figure 7a. O2 α absolute power dominated the ranking, contributing more than any other feature by a clear margin, and was followed by Fp1 θ relative power, Fz θ relative power, left occipital slow/fast ratio, F4 α relative power, and left temporal θ relative power. The 12 leading features were drawn exclusively from spectral feature families—relative spectral power, absolute spectral power, θ/α ratio, and slow/fast ratio—whereas the nonlinear complexity features (PE and DE) were not selected in any LOOCV fold. This indicates that spectral features provided the dominant discriminative information in the AHEPA cohort, though the presence of complexity features in the candidate pool ensured that the feature selection procedure was not restricted to a single feature type.

Across all 63 LOOCV folds, a total of 38 unique features were selected at least once. Of these, 3 features were selected in 60 or more folds (over 95%), and 11 features appeared in more than 50% of folds, indicating that the selection procedure identified a set of consistently selected features. Among the 12 leading features, 11 showed 100% coefficient direction consistency across all folds in which they were selected, and the remaining feature showed 96.6% direction consistency. The median coefficient of variation of the Ridge coefficients across folds was 0.12. Detailed per-feature stability metrics are provided in Supplementary Table S2. The complete selection frequency distribution is provided in Supplementary Figure S1.

The direction and subject-level heterogeneity of these contributions are visible in the beeswarm plot of Figure 7b. The directionality across features lined up with established AD-related EEG abnormalities. For O2 α absolute power, lower values carried positive SHAP and pushed predictions toward AD, consistent with the well-known reduction of posterior α-band activity in AD. Higher θ relative power (Fp1, Fz) and elevated slow/fast and θ/α ratios (left occipital, left temporal) carried positive SHAP and pushed predictions toward AD, matching the typical pattern of oscillatory slowing. The visible spread of points along each row indicates that the magnitude of these contributions varied considerably from subject to subject, as expected in a clinically heterogeneous sample.

Overall, the out-of-fold SHAP analysis indicates that AD–HC discrimination by the Ridge Logistic model was supported jointly by posterior α activity, frontal and midline θ power, and spectral ratio features, rather than by any single dominant feature. These results should be interpreted as fold-wise, model-level explanations of how the classifier used the available EEG features, and not as evidence that any individual feature alone constitutes a definitive AD biomarker.

### 3.5 Confounder analysis

To assess whether demographic variables could account for the observed classification performance, a three-model comparison was conducted under the same LOOCV framework. A demographic-only model using age and sex as features yielded an AUC of 0.58, near chance level, indicating that the age difference between groups (1.5 years) and the sex imbalance (AD: 67% female vs. HC: 41% female) carried little discriminative information in this comparison. When age and sex were added to the EEG feature pool (combined model), they were not selected in any LOOCV fold, and the combined model AUC (0.912) was identical to the EEG-only model. Thus, including the available age and sex variables did not materially improve discriminative performance in this LOOCV comparison. Technical variables were assessed descriptively: group mean recording durations were similar (AD: 13.5 min; HC: 13.8 min), all recordings had the same original sampling rate (500 Hz), additional epoch rejection was below 1%, and retained-epoch counts were at the 60-epoch cap for 62 of 63 participants. The near-invariant sampling-rate, rejection-rate, and retained-epoch measures did not provide sufficient variation for an informative covariate-model analysis; this descriptive assessment does not exclude residual technical confounding.

### 3.6 Microstate topographic dynamics

**Figure 8:**
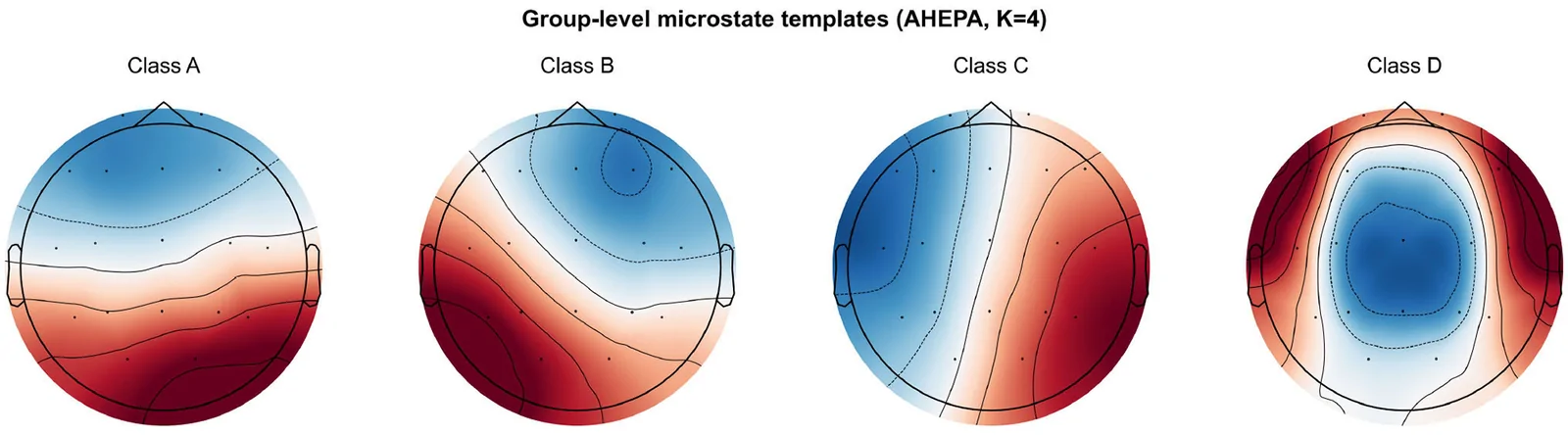
Group-level microstate topographic templates derived from polarity-invariant *k*-means clustering of GFP peak maps pooled across all 63 AHEPA subjects. The four templates are arranged as putative Classes A–D in order of descending explained variance. Color encodes relative scalp potential in arbitrary units (a.u.).

The classification analysis above relied on static spectral features that summarize EEG signals over time. To capture a different aspect of resting-state EEG, we additionally analyzed microstate topographic dynamics. Polarity-invariant *k*-means clustering of GFP peak maps pooled across all subjects (approximately 1,000 peaks per subject, 63,000 total) yielded four group-level templates (Figure 8). The *K* = 4 solution explained 56.4% of the total GFP peak variance (unweighted GEV). The resulting templates were labeled as putative Classes A–D in descending order of explained variance; because no spatial correlation with canonical templates was performed, these labels are provisional rather than established canonical assignments.

**Figure 9:**
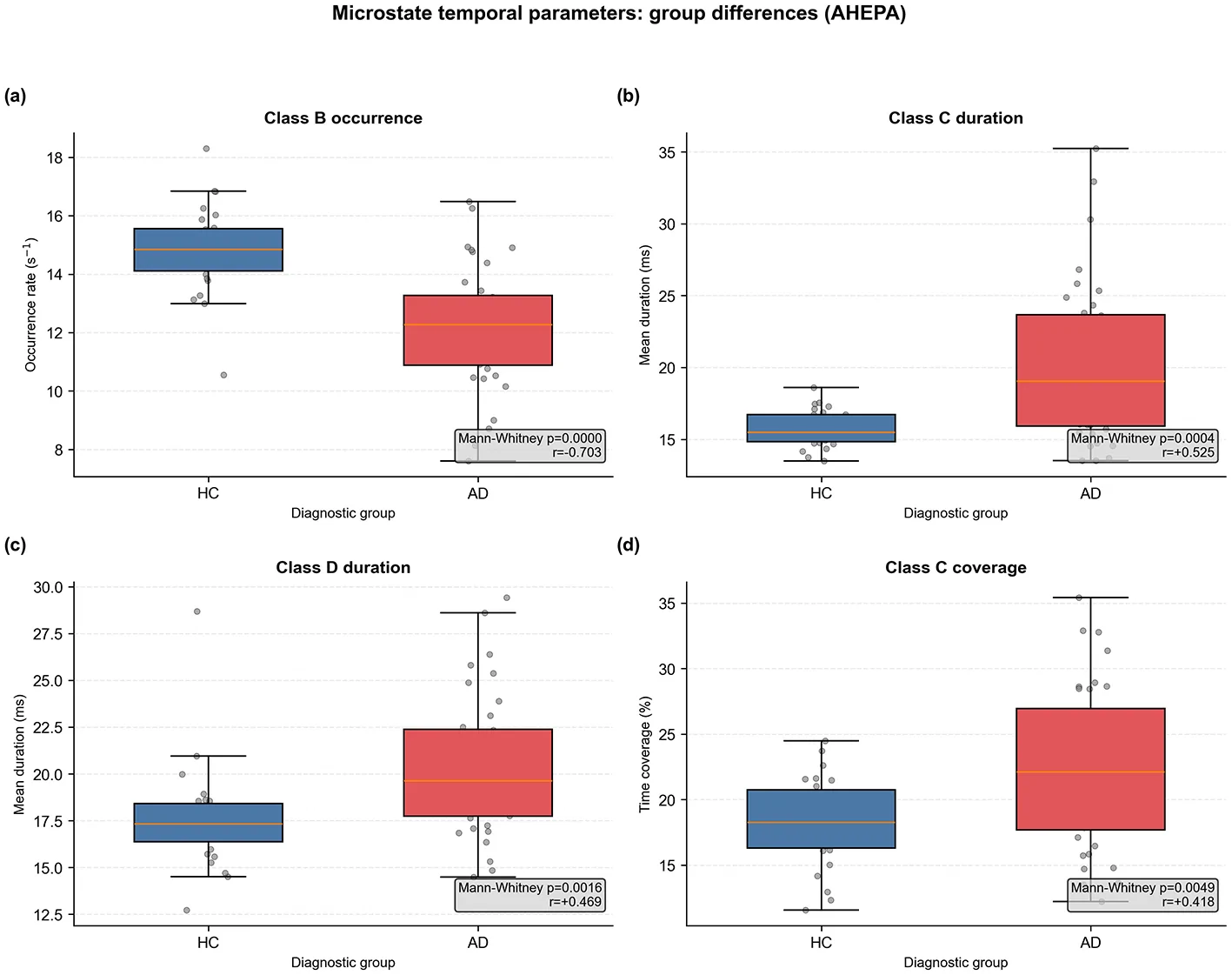
Subject-level microstate temporal parameters showing the largest group differences (AHEPA dataset). **(a)** Class B occurrence per second; **(b)** Class C mean duration; **(c)** Class D mean duration; **(d)** Class C time coverage. Boxplots show the median and interquartile range, with individual subject-level data points overlaid. Mann–Whitney *p*-values and rank-biserial correlations are annotated within each panel. HC, healthy controls (blue); AD, Alzheimer's disease (red).

Subject-level back-fitting revealed a redistribution of microstate temporal parameters in AD (Figure 9). A microstate consistent with a putative Class B assignment showed the largest group difference: its occurrence rate was reduced in AD (12.2 per second) relative to HC (14.8 per second; Mann–Whitney *p* < 0.001, rank-biserial *r* = −0.70), and its time coverage was correspondingly lower (AD: 24.1% vs. HC: 27.8%; *p* < 0.001, *r* = −0.52). A microstate consistent with a putative Class C assignment showed the opposite pattern, with increased mean duration in AD (20.2 ms vs. 15.7 ms; *p* < 0.001, *r* = +0.53) and increased coverage (AD: 22.6% vs. HC: 18.2%; *p* = 0.005, *r* = +0.42). A microstate consistent with a putative Class D assignment also showed increased duration in AD (20.3 ms vs. 17.6 ms; *p* = 0.002, *r* = +0.47). These subject-level group differences had medium-to-large rank-biserial effect sizes in this cohort.

To assess whether the principal temporal effects depended on a particular *K* = 4 initialization, clustering was repeated with 30 independent initializations. Across the primary solution and all 30 robustness solutions, reduced Class B occurrence, increased Class C duration, and increased Class D duration were significant in all 31 solutions. Their mean rank-biserial correlations were −0.57, +0.54, and +0.46, respectively. The mean absolute spatial correlations between the corresponding *K* = 4 templates and the reference templates were 0.94–0.98. In matched *K* = 3, *K* = 5, and *K* = 6 solutions, all three effects retained their respective directions and remained significant (*p* < 0.05; Supplementary Figure S2 and Supplementary Table S4). These checks support the reproducibility of the principal temporal effects within the present cohort, while the data-conditioned class labels remain provisional.

**Figure 10:**
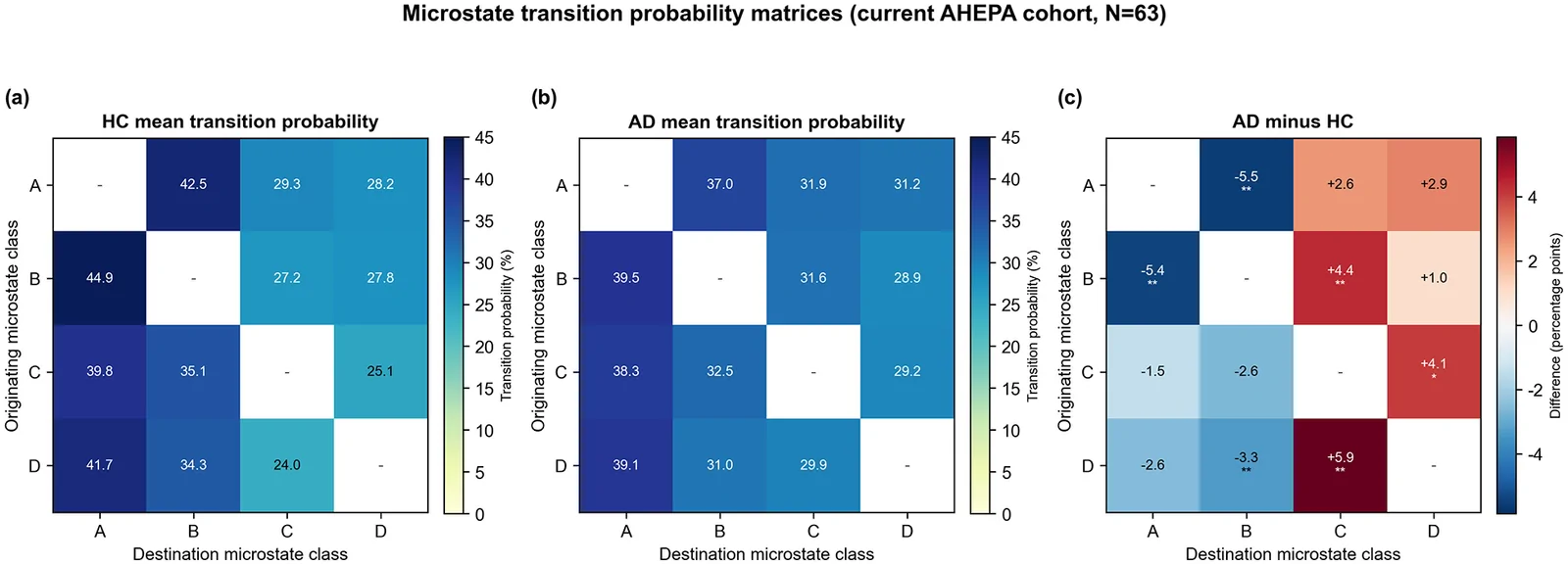
Microstate transition probability matrices in the current AHEPA cohort (63 subjects). **(a)** Mean subject-level transition probabilities in HC; **(b)** mean subject-level transition probabilities in AD; and **(c)** AD minus HC differences in percentage points. Rows indicate the originating class and columns the destination class. Self-transitions were excluded by construction. For each subject, off-diagonal transitions were calculated between consecutive run-length segments within each 5-s epoch and normalized by the row total; no transitions were counted across epoch boundaries. Asterisks in **(c)** denote Mann–Whitney group differences after Benjamini–Hochberg correction across the 12 off-diagonal transition tests (^*^pFDR < 0.05; ^**^pFDR < 0.01).

The transition analysis (Figure 10) showed six FDR-corrected group differences among the 12 off-diagonal transitions. Relative to HC, AD showed lower transition probabilities from Class A to Class B (37.0% vs. 42.5%; *p*_FDR_ = 0.002, *r* = −0.55), Class B to Class A (39.5% vs. 44.9%; *p*_FDR_ = 0.003, *r* = −0.51), and Class D to Class B (31.0% vs. 34.3%; *p*_FDR_ = 0.003, *r* = −0.50). Higher transition probabilities in AD were observed from Class B to Class C (31.6% vs. 27.2%; *p*_FDR_ = 0.005, *r* = +0.46), Class C to Class D (29.2% vs. 25.1%; *p*_FDR_ = 0.015, *r* = +0.40), and Class D to Class C (29.9% vs. 24.0%; *p*_FDR_ = 0.004, *r* = +0.48). These transition estimates describe adjacent run-length segments within the sampled epochs, use the primary *K* = 4 solution, and should be interpreted cautiously.

The temporal-parameter and transition analyses thus provide exploratory evidence of altered resting-state EEG topographic dynamics in AD. Similar microstate abnormalities have been reported across other neuropsychiatric conditions (Xue et al., 2024). Because these microstate features were never entered into the classifier, they constitute a classifier-independent complementary analysis alongside the spectral findings, consistent with the view that AD-related EEG alterations involve both oscillatory power changes and differences in the temporal organization of scalp potential dynamics.

## 4 Discussion

This study evaluated an interpretable resting-state EEG framework for distinguishing patients with AD from HC subjects in the publicly available AHEPA dataset, using subject-level spectral and nonlinear complexity features. The main findings were threefold. First, EEG-derived features showed favorable AD–HC discriminative performance under strict leave-one-subject-out cross-validation, with Ridge Logistic achieving an exploratory AUC of 0.912 across 63 subjects. The nearly identical performance of three of the four classifiers suggests that the discriminative information was not model-specific. Epoch-resampling sensitivity analysis yielded a mean AUC of 0.887 across 30 samples and an AUC of 0.917 using all accepted epochs, showing a favorable but sample-dependent classification signal. A demographic-only model (age + sex) yielded near-chance performance (AUC = 0.58), and including these variables did not materially improve discriminative performance in the combined LOOCV comparison. Second, the out-of-fold SHAP analysis indicated that the classification model relied on multiple complementary EEG feature families, including posterior alpha activity, frontal and temporal theta power, spectral ratio features, and slow/fast ratio features, rather than a single dominant marker. Eleven of the 12 leading features showed consistent coefficient direction across all folds, supporting the stability of the identified EEG patterns. Third, complementary microstate analysis revealed altered resting-state scalp topographic dynamics in AD, characterized by reduced occurrence of a putative Class B template, increased duration of putative Class C and Class D templates, and FDR-corrected differences in six off-diagonal transition probabilities. The three principal temporal effects were reproduced across repeated initializations and matched alternative *K* solutions. Together, these findings suggest that AD-related EEG alterations involve multiple levels of information, including changes in oscillatory power and the temporal organization of scalp potential configurations.

A key methodological feature of this study is the use of subject-level validation with fold-wise feature selection and model fitting. In small-sample EEG classification studies, segment-level cross-validation can lead to overly optimistic performance estimates because epochs from the same subject may appear in both the training and testing sets. To reduce this risk, the present study treated each subject, rather than each epoch, as the independent validation unit. Within each leave-one-subject-out fold, missing-value imputation, feature ranking, redundancy pruning, feature standardization, and classifier fitting were performed using only the training subjects, while the held-out subject was used exclusively for evaluation. This design provides a more conservative estimate of subject-level generalization than segment-wise validation. In addition, the comparable performance observed across multiple linear classifiers suggests that the discriminative information was not driven by a single model specification, although Ridge Logistic provided the most favorable balance between predictive performance and interpretability in the present dataset.

However, an important methodological limitation must be acknowledged: the number of selected features (*k* = 12) and the use of Ridge Logistic for SHAP visualization were identified from the same global LOOCV procedure used to report the final AUC. A nested cross-validation design, in which *k* and the classifier are selected within each outer training fold, would provide a less optimistic estimate of generalization performance. In the present study, nested validation was not performed because of the small sample size (*N* = 63), which would render the inner cross-validation estimates highly unstable. Consequently, the reported AUC of 0.912 should be regarded as an exploratory upper-bound estimate rather than a confirmed performance level, and independent evaluation remains necessary before any clinical interpretation. The epoch-resampling analysis further shows that the fixed-seed AUC is not a unique estimate: the 30-sample mean was 0.887 with moderate variation, although use of all accepted epochs yielded AUC = 0.917. High-frequency selected features were accompanied by variation in secondary selected features across resamples.

The out-of-fold SHAP analysis further helped clarify the feature-level basis of the classification model. The leading explanatory features included occipital alpha absolute power, frontal and midline theta relative power, and occipital and temporal slow/fast and theta/alpha ratio features. All 12 leading features belonged to the spectral domain (band power, spectral ratios), whereas nonlinear complexity features (PE, DE) were not selected in any fold, suggesting that spectral features provided the dominant discriminative information in this cohort. Importantly, the SHAP analysis was performed in an out-of-fold manner: each subject's explanation was derived from the fold-specific model trained without that subject, thereby maintaining consistency with the validation strategy used for performance evaluation. These SHAP results reflect model-level attributions of feature importance rather than independent neurophysiological validation. The highlighted features should be interpreted as fold-wise, model-identified discriminative patterns in this cohort, not as confirmed AD biomarkers. The heterogeneous SHAP distributions across subjects also indicate that feature contributions varied at the individual level, which is expected in clinical EEG data with limited sample size and inter-subject variability.

The microstate analysis provided an additional dynamic perspective independent of the classification model. The pooled four-class microstate solution yielded putative Class A–D topographic templates. Group comparisons revealed altered temporal parameters, with the strongest effect being reduced occurrence of the putative Class B microstate in AD (rank-biserial *r* = −0.70) and increased duration of the putative Class C microstate (*r* = +0.53). The current-cohort transition analysis further identified six FDR-corrected differences in off-diagonal switching probabilities. Across the primary solution and 30 additional *K* = 4 initializations, the three principal temporal effects were significant in all 31 solutions and their matched template correlations were high on average. The same effects retained their directions and statistical significance in matched *K* = 3, *K* = 5, and *K* = 6 solutions. These results support within-cohort robustness of the main temporal findings, but do not establish externally validated canonical class identities. Although canonical EEG microstate classes have been linked to large-scale resting-state networks in previous studies, the A–D labels in the present study were assigned by variance ordering rather than by spatial correlation with canonical templates or source-localized validation. Therefore, interpretations of these templates in terms of specific functional networks should be considered tentative. The transition estimates were derived within epochs and from the primary *K* = 4 solution, and should not be interpreted as continuous-recording dynamics.

Several limitations should be acknowledged. First, the classification pipeline used a non-nested validation design: both the feature count (*k* = 12) and the use of Ridge Logistic for SHAP visualization were identified from the global LOOCV profile. Although adjacent *k* values produced comparable AUCs (*k* = 10: 0.896; *k* = 14: 0.900), the reported AUC should be regarded as an exploratory upper-bound estimate rather than a validated performance level. Second, the AHEPA dataset includes clinically diagnosed probable AD without biomarker confirmation via PET, CSF, or MRI. Third, the analysis evaluates one public, single-site cohort; the sample size remains limited (*N* = 63) relative to the initial feature space (*N* = 373 features), and the AD and HC groups represent clinical extremes (mean MMSE 17.75 vs. 30), so performance in populations with milder cognitive impairment (e.g., MCI or early-stage AD) remains untested. Fourth, the present study used the preprocessed AHEPA derivatives (ASR + ICA); results may differ if raw data were processed with a different artifact-rejection pipeline. Fifth, clinical variables beyond age, sex, and MMSE (e.g., medication status, comorbidities, disease duration at the individual level) were not available for inclusion as covariates. Finally, the analysis was based on 19-channel clinical EEG recordings, which limits spatial resolution. Future work should validate this framework in multi-center cohorts with broader cognitive severity ranges and explore whether spectral, complexity, and microstate features can be combined with cognitive assessments or multimodal biomarkers to monitor disease progression.

## 5 Conclusion

In summary, this study evaluated an interpretable resting-state EEG framework for distinguishing patients with clinically diagnosed probable Alzheimer's disease from healthy control subjects in the publicly available AHEPA dataset (63 subjects). Under leave-one-subject-out cross-validation with fold-wise feature selection and model fitting, Ridge Logistic achieved an exploratory AUC of 0.912 (without nested validation) and an accuracy of 0.857. Across epoch resamples, mean AUC was 0.887 ± 0.020, and analysis of all accepted epochs yielded AUC = 0.917, indicating a favorable but sample-dependent signal. A demographic-only model yielded near-chance performance (AUC = 0.58), and including the available age and sex variables did not materially improve discrimination in this LOOCV comparison. Out-of-fold SHAP analysis indicated that the classification model relied on spectral EEG features, including posterior alpha activity, frontal and midline theta power, and spectral ratio features, rather than a single dominant marker. Eleven of the 12 leading features showed 100% coefficient direction consistency across LOOCV folds. In parallel, classifier-independent microstate analysis revealed altered temporal parameters and off-diagonal transition probabilities in AD; the three principal temporal effects were robust across repeated initializations and matched alternative *K* solutions. These findings suggest that resting-state EEG provides non-invasive and interpretable information for characterizing AD-related functional alterations. These findings should be considered exploratory pending validation in larger, multi-center cohorts with broader cognitive severity ranges.

## Data Availability

The raw data supporting the conclusions of this article will be made available by the authors, without undue reservation.
