# Supplementary material for: Explainable machine learning for Alzheimer's disease characterization using small-sample EEG data
Source: Front Aging Neurosci. 2026 Sep 7;18:1900857. doi: 10.3389/fnagi.2026.1900857 (PMC13590570; doi:10.3389/fnagi.2026.1900857)
Supplement: Supplementary file 1 [file Data_Sheet_1.pdf]

Supplementary Material

Supplementary Table S1. Subject-level epoch quality-control and sampling summary

Subject	Group	Initial 5-s epochs	Rejected	Rejected (%)	Retained
sub-001	AD	107	0	0.0%	60
sub-002	AD	146	0	0.0%	60
sub-003	AD	49	0	0.0%	49
sub-004	AD	129	0	0.0%	60
sub-005	AD	148	0	0.0%	60
sub-006	AD	114	0	0.0%	60
sub-007	AD	141	0	0.0%	60
sub-008	AD	146	1	0.7%	60
sub-009	AD	110	0	0.0%	60
sub-010	AD	244	0	0.0%	60
sub-011	AD	141	3	2.1%	60
sub-012	AD	164	2	1.2%	60
sub-013	AD	155	0	0.0%	60
sub-014	AD	174	2	1.1%	60
sub-015	AD	168	0	0.0%	60
sub-016	AD	182	6	3.3%	60
sub-017	AD	156	0	0.0%	60
sub-018	AD	157	0	0.0%	60
sub-019	AD	171	0	0.0%	60
sub-020	AD	161	0	0.0%	60
sub-021	AD	172	0	0.0%	60
sub-022	AD	152	4	2.6%	60
sub-023	AD	154	1	0.6%	60
sub-024	AD	139	0	0.0%	60
sub-025	AD	124	0	0.0%	60
sub-026	AD	167	0	0.0%	60
sub-027	AD	153	1	0.7%	60
sub-028	AD	151	0	0.0%	60
sub-029	AD	135	0	0.0%	60
sub-030	AD	98	3	3.1%	60
sub-031	AD	218	2	0.9%	60
sub-032	AD	147	0	0.0%	60
sub-033	AD	129	0	0.0%	60
sub-034	AD	181	0	0.0%	60
sub-035	AD	136	0	0.0%	60
sub-036	AD	156	0	0.0%	60
sub-037	HC	143	0	0.0%	60
sub-038	HC	166	1	0.6%	60
sub-039	HC	158	0	0.0%	60
sub-040	HC	181	1	0.6%	60
sub-041	HC	165	0	0.0%	60
sub-042	HC	180	1	0.6%	60
sub-043	HC	153	0	0.0%	60
sub-044	HC	164	0	0.0%	60
sub-045	HC	158	1	0.6%	60
sub-046	HC	138	0	0.0%	60
sub-047	HC	149	2	1.3%	60
sub-049	HC	144	0	0.0%	60
sub-050	HC	151	1	0.7%	60
sub-051	HC	138	0	0.0%	60

Subject	Group	Initial 5-s epochs	Rejected	Rejected (%)	Retained
sub-052	HC	140	0	0.0%	60
sub-053	HC	144	0	0.0%	60
sub-054	HC	155	0	0.0%	60
sub-055	HC	150	0	0.0%	60
sub-056	HC	146	0	0.0%	60
sub-057	HC	147	0	0.0%	60
sub-058	HC	139	0	0.0%	60
sub-059	HC	145	0	0.0%	60
sub-060	HC	137	1	0.7%	60
sub-061	HC	148	0	0.0%	60
sub-063	HC	148	0	0.0%	60
sub-064	HC	157	0	0.0%	60
sub-065	HC	164	0	0.0%	60

AD: median initial epochs 152 (range 49–244), median rejected 0 (range 0–6), median retained 60 (range 49–60). HC: median initial epochs 149 (range 137–181), median rejected 0 (range 0–2), median retained 60 (range 60–60). Retained epochs were selected randomly without replacement from accepted epochs using fixed seed 2026, capped at 60 per subject.

Supplementary Figure S1. Feature selection frequency across LOOCV folds

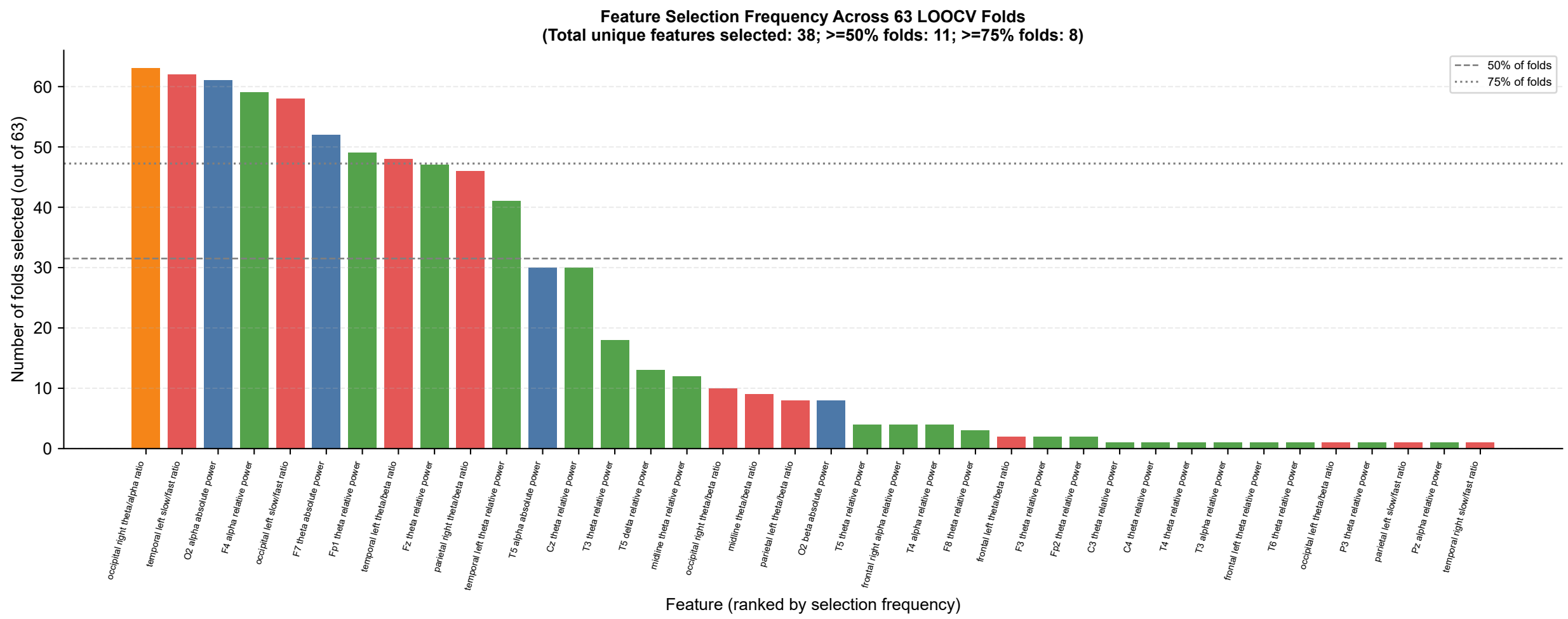

Selection frequency distribution across the 63 leave-one-subject-out cross-validation folds for all 38 features that were selected at least once. Bars are ordered by selection frequency (descending). Three features were selected in 60 or more folds (over 95%), and 11 features appeared in more than 50% of folds. The remaining low-frequency features were selected in only a few folds.

Supplementary Table S2. Per-feature stability metrics for the 12 features ranked by selection-weighted mean absolute SHAP value

Feature	Selected	Freq.	Direction	Coef. (mean ± SD)	Mean |SHAP| ± SD	SHAP CV
O2 α absolute power	61/63	96.8%	100.0%	-1.04 ± 0.14	0.77 ± 1.08	1.39
Fp1 θ relative power	49/63	77.8%	100.0%	$+0.70 \pm 0.06$	0.61 ± 0.50	0.81
Fz θ relative power	47/63	74.6%	100.0%	$+0.64 \pm 0.06$	0.60 ± 0.35	0.59
L occipital slow/fast ratio	58/63	92.1%	100.0%	$+0.56 \pm 0.05$	0.45 ± 0.42	0.92
F4 α relative power	59/63	93.7%	96.6%	$+0.48 \pm 0.15$	0.41 ± 0.25	0.62
L temporal θ rel. power	41/63	65.1%	100.0%	$+0.52 \pm 0.06$	0.51 ± 0.34	0.68
R occipital θ/α ratio	63/63	100.0%	100.0%	$+0.35 \pm 0.07$	0.29 ± 0.25	0.85
L temporal θ/β ratio	48/63	76.2%	100.0%	$+0.36 \pm 0.06$	0.28 ± 0.28	0.99
L temporal slow/fast ratio	62/63	98.4%	100.0%	$+0.26 \pm 0.08$	0.19 ± 0.19	1.00
T3 θ relative power	18/63	28.6%	100.0%	$+0.90 \pm 0.08$	0.52 ± 0.27	0.52
R parietal θ/β ratio	46/63	73.0%	100.0%	$+0.22 \pm 0.05$	0.19 ± 0.20	1.08
T5 α absolute power	30/63	47.6%	100.0%	-0.70 ± 0.06	0.26 ± 0.20	0.78

L, left; R, right; Coef., Ridge Logistic coefficient (logit scale); |SHAP|, absolute out-of-fold SHAP value when selected; SD, standard deviation; CV, coefficient of variation (SD/mean). Direction is the proportion of selected folds in which the coefficient sign matches the majority direction. The median coefficient of variation of the Ridge coefficients across the 12 features was 0.12.

Supplementary Table S3. Epoch-sampling sensitivity analysis of the Ridge Logistic LOOCV model

Epoch analysis	AUC	Accuracy	Sensitivity	Specificity
All accepted epochs	0.917	0.841	0.750	0.963
30 balanced random samples	0.887 ± 0.020 [0.829–0.921]	0.811 ± 0.024 [0.762–0.857]	0.747 ± 0.023 [0.694–0.806]	0.896 ± 0.036 [0.815–0.963]

For each of 30 independently balanced random samples (seeds 2026–2055), up to 60 accepted epochs were drawn without replacement for each subject, all 373 subject-level features were recomputed, and the full fold-wise Ridge Logistic LOOCV pipeline was rerun. Values are mean ± SD [minimum–maximum]. The all-accepted-epochs row uses every accepted epoch for each subject. The seed-2026 random sample reproduces the primary analysis (AUC 0.912; accuracy 0.857).

Supplementary Figure S2. Microstate initialization and K-sensitivity robustness

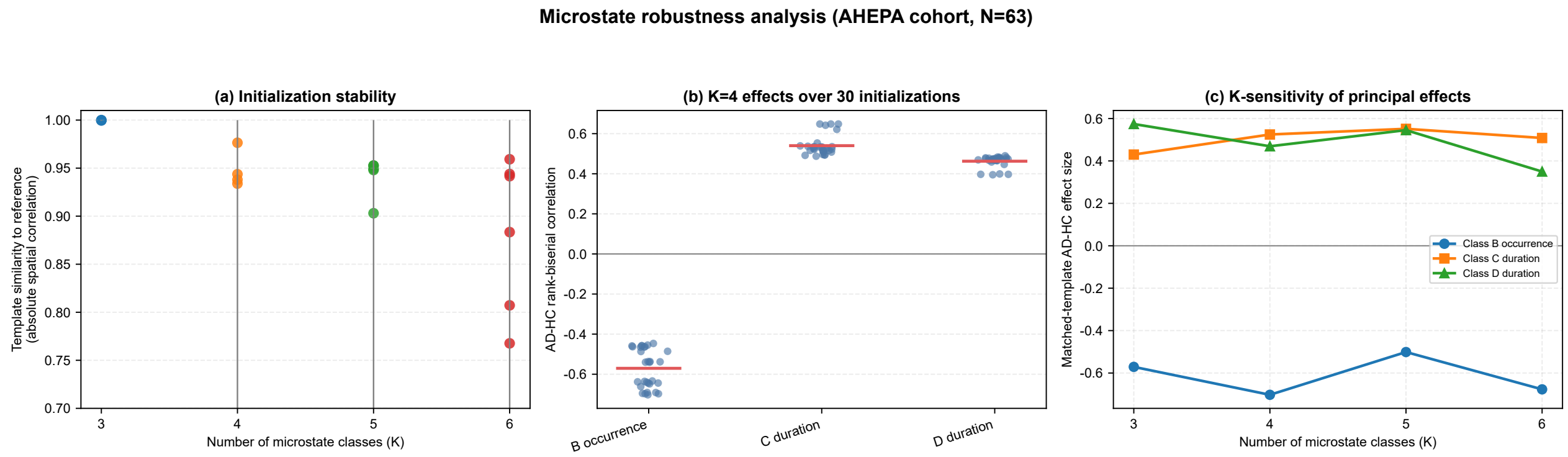

Thirty independent polarity-invariant k-means initializations were evaluated at each of $K = 3, 4, 5, 6$. Panel (a) summarizes absolute spatial correlations between each aligned template and its reference solution. Panel (b) shows the rank-biserial group effect for the three primary $K = 4$ temporal findings across the 30 robustness initializations. Panel (c) reports the group effects for templates matched to the primary $K = 4$ topographies across alternative values of K.

Supplementary Table S4. Microstate robustness across repeated initialization and alternative K solutions

Matched temporal effect	$K = 3$	$K = 4$	$K = 5$	$K = 6$
Class B occurrence	−0.571 ($p < 0.001$)	−0.703 ($p < 0.001$)	−0.501 ($p < 0.001$)	−0.677 ($p < 0.001$)
Class C duration	+0.430 ($p = 0.004$)	+0.525 ($p < 0.001$)	+0.551 ($p < 0.001$)	+0.508 ($p < 0.001$)
Class D duration	+0.574 ($p < 0.001$)	+0.469 ($p = 0.002$)	+0.545 ($p < 0.001$)	+0.350 ($p = 0.019$)

Entries are AD–HC rank-biserial correlations with two-sided Mann–Whitney p values for the template in each alternative-K solution having the highest absolute spatial correlation with the corresponding primary $K = 4$ template. For the primary $K = 4$ solution plus 30 independent robustness initializations, Classes A–D had mean absolute template correlations of 0.976, 0.944, 0.934, and 0.938 relative to the primary solution, respectively; mean GEV was 0.5638 ± 0.0005. Class B occurrence, Class C duration, and Class D duration were significant in all 31 examined $K = 4$ solutions, with rank-biserial correlations of −0.575 ± 0.098, +0.540 ± 0.048, and +0.463 ± 0.027, respectively.
